# Noticing cigarette health warnings and support for new health warnings among non-smokers in China: findings from the International Tobacco Control project (ITC) China survey

**DOI:** 10.1186/s12889-017-4397-2

**Published:** 2017-05-19

**Authors:** Zejun Li, Tara Elton Marshall, Geoffrey T. Fong, Anne Chiew Kin Quah, Guoze Feng, Yuan Jiang, Sara C. Hitchman

**Affiliations:** 1 Department of Basic and Clinical Neuroscience, Institute of Psychiatry, Psychology, and Neuroscience, King’s College London, London, England; 2 Social and Epidemiological Research Department, Centre for Addiction and Mental Health, London, Canada; 3 Department of Epidemiology and Biostatistics, Western University, London, Canada; 4 Dalla Lana School of Public Health, University of Toronto, Toronto, Canada; 5 Department of Psychology, University of Waterloo, Waterloo, Canada; 6 School of Public Health and Health Systems, University of Waterloo, Waterloo, Canada; 7 Ontario Institute for Cancer Research, Toronto, ON Canada; 8 Tobacco Control Office, Chinese Center for Disease Control and Prevention, Beijing, China; 9 Department of Addictions, Institute of Psychiatry, Psychology, and Neuroscience, King’s College London, London, England; 10 UK Centre for Tobacco and Alcohol Studies, Nottingham, UK; 11 Addictions Department, Addictions Sciences Building, Institute of Psychiatry, Psychology and Neuroscience (IoPPN), King’s College London, 4 Windsor Walk, Denmark Hill, London, SE5 8BB England

**Keywords:** China, Cigarette health warning labels, Non-smokers, Smoking, Health promotion

## Abstract

**Background:**

Health warnings labels (HWLs) have the potential to effectively communicate the health risks of smoking to smokers and non-smokers, and encourage smokers to quit. This study sought to examine whether non-smokers in China notice the current text-only HWLs and whether they support adding more health information and including pictures on HWLs.

**Methods:**

Adult non-smokers (*n* = 1324) were drawn from Wave 4 (September 2011–November 2012) of the International Tobacco Control (ITC) China Survey. The proportion of non-smokers who noticed the HWLs, and supported adding more health information and pictures to the HWLs was examined. Additionally, the relation between non-smokers’ demographic characteristics, including whether they had a smoking partner, their number of smoking friends, and noticing the HWLs and support for adding health information and pictures was examined. Because the HWLs changed during the survey period (April 2012), differences between non-smokers who completed the survey before and after the change were examined.

**Results:**

12.2% reported they noticed the HWLs often in the last month. The multivariate model, adjusting for demographics showed that respondents with a smoking partner (OR = 2.41, 95% CI 1.42–4.13, *p* = 0.001) noticed the HWLs more often. 64.8% of respondents agreed that the HWLs should have more information, and 80.2% supported including pictures. The multivariate model showed that non-smokers who completed the survey after the HWLs were implemented (OR = 0.63, 95% CI 0.40–0.99, *p* = 0.04) were less likely to support adding more health information. The multivariate model showed a significant relation between having a smoking partner and supporting pictorial HWLs (OR = 2.03, 95% CI 1.24–3.33, *p =* 0.005).

**Conclusions:**

The findings indicate that the Chinese HWLs are noticed by a minority of non-smokers and that non-smokers strongly support strengthening the Chinese warning labels with more health information and pictures. Additionally, because the HWLs are noticed more often by non-smokers with a smoking spouse/partner, HWLs could be used to communicate the dangers of smoking and secondhand smoke exposure to non-smokers.

## Background

Globally, smoking is the leading cause of preventable death [1]. In 2015, approximately 49.3% of males and 2.0% of females 15 years and older smoked in China [2]. Due to the high prevalence of smoking, approximately 72.4% of non-smokers in China were exposed to secondhand smoke at least weekly, with 38% reporting daily exposure [3]. This is cause for concern as secondhand smoke exposure can cause significant health problems including adult heart disease, and lung diseases in children [3–5].

**Fig. 1:**
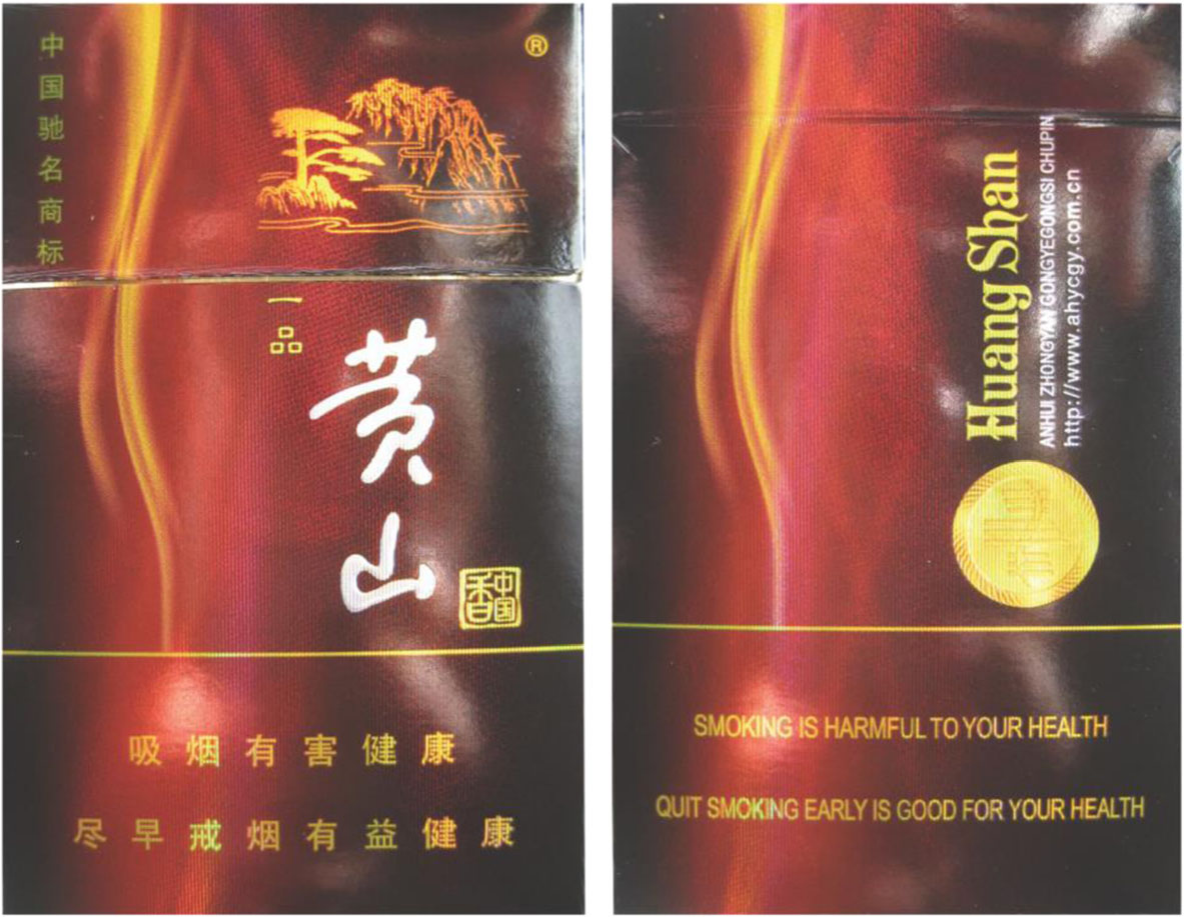
2009 Chinese Health Warning Labels, left (*front of pack*), right (*back of pack*)

**Fig. 2:**
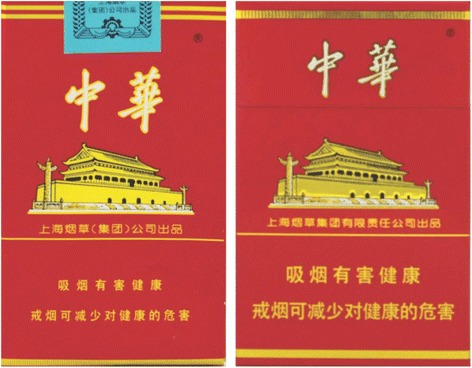
Chinese Health Warning Labels, left (2009 warning labels), right (2012 larger warning labels)

To reduce the harms from smoking, China ratified the World Health Organization’s (WHO) Framework Convention on Tobacco Control (FCTC) in 2005 and took steps to implement the FCTC [6]. In January 2009, China changed the text-based HWLs that appeared on the side of the pack and replaced them with HWLs that covered 30% of the front and back bottom of the package. The warnings messages included two sets of general warnings (e.g., one of the two sets: ‘Smoking is harmful to your health’ and ‘Quitting smoking early is good for your health’). Interestingly, the HWLs appearing on the back were printed in English and were identical to the Chinese characters on the front (Fig. 1) [6]. In April 2012, the Chinese HWLs were changed again. The English text on the back was changed to Chinese characters and the minimum text font size was increased from 2.0 mm to 4.0 mm (Fig. 2) [7]. Although the current HWLs in China meet the FCTC minimum requirements, they don’t meet the FCTC guidelines for HWLs, which suggest that HWLs cover at least 50% of the pack and use pictures. Large pictorial health warnings have been shown to be more effective than smaller text-only HWLs and are important for informing people about the health risks of smoking and encouraging smokers to quit [8–12].

A previous study of the HWLs in China and Malaysia showed that when the HWLs in China were changed from text-only to larger text-only HWLs the proportion of smokers who noticed HWLs ‘often’ increased from 41.6 to 44.7%, while in Malaysia, where pictorial HWLs were introduced, smokers who noticed HWLs ‘often’ or ‘very often’ increased from 54.4 to 67.0% [8]. However, there have been no studies to date examining noticing HWLs among non-smokers in China. Thus, it is worth considering the impact of HWLs on non-smokers. HWLs may warn non-smokers about the health effects of smoking and secondhand smoke, the health risks of smoking among their smoking family and friends, and may deter smoking uptake. HWLs may be particularly important for non-smokers in China for three main reasons: (1) Chinese non-smokers have a high rate of exposure at home to secondhand smoke and need to be warned about its harms [3–5], (2) China is a collectivistic culture and it is possible that non-smoking family members or close others could be a powerful influence on encouraging their close others to quit [13, 14], and (3) the low rates of smoking among women need to be maintained and HWLs could warn women about the health risks of starting to smoke. Thus, the aim of this study was to examine: (1) whether non-smokers in China notice the HWLs on cigarettes, (2) whether non-smokers with smoking spouses or partners and friends notice the warnings more often, and (3) whether non-smokers support adding more information and pictures to the current text only HWLs.

## Methods

### Survey

Data are from the International Tobacco Control (ITC) China Survey Wave 4. The ITC China Survey is a cohort survey of adult smokers and non-smokers. The ITC China Survey used a multistage cluster sampling design to recruit 800 smokers and 200 non-smokers in each survey city: Stage 1 Jie Dao (street district), Stage 2 Ju Wei Hui (residential blocks) and Stage 3 at the household level. The survey used quantitative methods and was conducted via face-to-face interviews in seven cities: Beijing, Shanghai, Changsha, Guangzhou, Yinchuan, Kunming, and Shenyang according to the geographical representation and levels of economic development difference [15]. Overall, there were 1324 non-smokers in the Wave 4 data collection. Further details about the methodology and survey design are available elsewhere [15, 16].

### Measures

#### Key outcome variables

*Noticing HWLs*. ‘In the last month, how often, if at all, have you noticed the health warnings on cigarette packages?’ Responses were dichotomized as “often” (‘often’) vs. “less than often” (‘once in a while,’ ‘never,’ ‘refused’, or ‘don’t know.’)

*Support for more health information on HWLs.* ‘Do you think that cigarette packages should have more health information than they do now, less, or about the same amount as they do now?’ Responses were dichotomized as “does not support” (‘less information’, ‘same information’ ‘refused’, ‘don’t know’) vs. “support” (‘more health information’).

*Support for including pictures on HWLs.* ‘Would you support or oppose the government including pictures as part of the health warning on cigarette packs?’ Responses were dichotomized as “does not support” (‘strongly oppose, ‘oppose’, ‘refused’, ‘don’t know’) vs. “support” (‘strongly support’, ‘support’).

*Surveyed Before/After new HWLs implemented*. Because the new HWLs were introduced while the survey was in the field, a variable was coded to indicate surveyed “before” vs. “after” the new HWLs were implemented.

#### Key independent variables

##### Partner and Friend Smoking

*Smoking spouse/partner*. Respondents with a partner were asked: ‘Does your partner or spouse smoke?’ Responses were coded as “no smoking spouse/partner” = ‘no’; “have smoking spouse/partner” = ‘yes’; “no spouse/partner” = ‘refused’, ‘don’t know’. Respondents who were not married or not living with their partners were coded as “no spouse/partner”.

*Number of smoking friends*. ‘Of the five closest friends or acquaintances (not including family members) that you spend time with on a regular basis, how many of them are smokers?’ Responses were coded as “0, 1, 2, 3, 4, 5”.

#### Demographic variables

Demographic variables included: city, ethnicity (Han nationality, other nationality), age (18–39, 40–54 and 55+), gender (male, female), monthly household income (Low/Medium: <¥3000, High:≧¥3000), and education (Low/Medium = no education, elementary school, junior/senior high school, High = college, university or higher.

### Statistical analysis

The data were analysed using Stata/MP 14.0 software for Mac. Descriptive and sample characteristics were examined. Bivariate and multiple logistic regression models (controlling for demographics) were used to examine the relation between the outcome, noticing the HWLs often vs. less often in the last month, and respondents’ number of smoking friends, whether respondents had a smoking spouse/partner, and whether they were surveyed before vs. after new HWLs were introduced. To examine differences in support for adding more information and including pictures on Chinese HWLs, the predicted outcome of a logistic regression analysis was set to “support for more information” versus “no support” and “support for including pictures” versus “no support”. These analyses were controlled for demographics, noticing HWLs in the last month, number of smoking friends, having a smoking partner and surveyed before versus after new HWLs were introduced. All analyses were conducted on weighted data.

## Results

### Demographic characteristics

The majority (92.7%) of the respondents were Han, female (62.6%), over the age of 55 (48.2%), had high household income (63.2%), and had low or medium education (67.5%). 31.34% had a smoking spouse/partner and most had no smoking friends (30.9%). Very few respondents had 4 (5.2%) or 5 (8.5%) smoking friends (See Table 1).

**Table 1:** Characteristics associated with whether non-smokers noticed health warning labels often vs. less than often in the last month (*N* = 1324)

	*N*	%	Noticed HWLs Often (%)	*p*	OR	95% CI
City						
Beijing	202	15.25%	3.49%	ref	ref	ref
Shenyang	187	14.12%	8.16%	0.192	2.45	0.63–9.52
Shanghai	196	14.80%	3.75%	0.831	1.17	0.28–4.87
Changsha	180	13.60%	17.78%	0.033^a^	4.72	1.14–19.62
Guangzhou	191	14.43%	11.31%	0.015^a^	4.07	1.33–12.46
Kunming	180	13.60%	19.57%	0.01^a^	5.02	1.48–17.05
Yinchuan	188	14.20%	23.04%	0.002^a^	7.12	2.12–23.92
Ethnicity						
Others	97	7.33%	28.65%	ref	ref	ref
Han nationality	1227	92.67%	11.08%	0.007^a^	0.40	0.21–0.77
Gender						
Male	495	37.39%	12.34%	ref	ref	ref
Female	829	62.61%	12.08%	0.110	0.64	0.37–1.11
Age (years)						
18–39	248	18.73%	21.27%	ref	ref	ref
40–54	438	33.08%	13.24%	0.097	0.60	0.32–1.10
55+	638	48.19%	8.72%	0.014^a^	0.48	0.27–0.86
Household Income per month (yuan)						
Low/Medium	487	36.78%	11.31%	ref	ref	ref
High	837	63.22%	12.64%	0.100	1.56	0.92–2.66
Education						
Low/Medium	894	67.52%	11.06%			
High	430	32.48%	14.90%	0.978	1.00	0.51–1.93
Smoking spouse/partner						
No smoking spouse/partner	721	54.46%	10.28%	ref	ref	ref
Have smoking spouse/partner	415	31.34%	19.13%	0.002^a^	2.41	1.42–4.13
No spouse/partner	188	14.20%	8.15%	0.763	0.90	0.43–1.86
Number of smoking friends						
0	409	30.89%	10.57%	ref	ref	ref
1	238	17.98%	10.35%	0.617	0.80	0.33–1.95
2	264	19.94%	10.12%	0.204	0.60	0.27–1.33
3	232	17.52%	13.72%	0.821	0.92	0.46–1.84
4	69	5.21%	9.74%	0.647	0.69	0.14–3.39
5	112	8.46%	24.35%	0.333	1.59	0.62–4.11
Surveyed before/after new HWLs apply						
Before	1100	83.08%	10.40%	ref	ref	ref
After	224	16.92%	19.50%	0.628	1.17	0.61–2.25

*CI* Confidence interval; Significant levels are indicated as follows: *p < 0.05; **p < 0.001,***p < 0.0001

OR, Odd Ratio of noticed health warning labels (0, refused/don’t know/once a while noting warning labels in last month; 1, often noticed labels in last month)

Survey before/after new HWLs apply, Before (before 01/04/2012); After (after 01/04/2012)

Notice HWLs often, Respondents who reported they notice HWLs often in the last month

^a^The percentage are weighted and the frequencies are unweighted

### Noticing HWLs

12.2% of respondents reported noticing HWLs often, 20.1% reported noticing HWLs ‘once in a while’ and 67.7% reported ‘never’ noticing HWLs in the last month. In the bivariate logistic regression analysis, non-smokers who had a smoking spouse/partner (OR = 2.06, 95% CI 1.24–3.42 *p* = 0.006), with 5 vs. 0 smoking friends (OR = 2.72, 95% CI 1.09–6.82, *p* = 0.033), and surveyed after the new HWLs were implemented (OR = 2.09, 95% CI 1.20–3.63, *p* = 0.010) were more likely to notice the HWLs more often. In multivariate logistic regression analysis, non-smokers who had a smoking spouse/partner (OR = 2.41, 95% CI 1.42–4.13, *p =* 0.002) noticed HWLs more often than those who did not have smoking spouse/partner. Additional findings included, older and Han ethnicity non-smokers were less likely to notice the warnings. There were also unexpected city differences in noticing the HWLs (See Table 1).

### Support for strengthening the HWLs with more information and pictures

About 64.8% of respondents agreed that cigarette packages should have more health information and 80.2% supported government including pictures as part of the HWLs. In the bivariate logistic regression model, for non-smokers who supported adding more health information, there were no significant differences in whether they had a smoking spouse/partner or not, smoking friends and whether they were interviewed before or after the new HWLs were implemented. In the multivariate logistic regression analysis, non-smokers who were interviewed after the new HWLs were implemented (OR = 0.63, 95% CI 0.40–0.99, *p* = 0.04) were less likely to support adding more health information (See Table 2). There were also city differences. In the bivariate logistic regression analysis, non-smokers who noticed HWLs in the last month (OR = 2.52, 95% CI 1.35–4.71, *p* = 0.004) and who had smoking spouse (OR = 2.12, 95% CI 1.34–3.38, *p* = 0.002) were more likely to support including pictures on the HWLs. In the multivariate logistic regression analyses, non-smokers who had a smoking spouse or partner (OR = 2.03, 95% CI 1.24–3.33, *p* = 0.005) were more likely to support including pictures in HWLs, with no differences between those surveyed before or after the new HWLs were implemented (See Table 3). There were also city differences.

**Table 2:** Characteristics associated with whether non-smokers supported adding more information to cigarette packages (*N* = 1324)

	*N*	Support more information (%)	*p*	OR	95% CI
City					
Beijing	202	69.78%	ref	ref	ref
Shenyang	187	66.02%	0.797	0.94	0.57–1.54
Shanghai	196	55.93%	0.111	0.49	0.20–1.18
Changsha	180	52.53%	0.009^a^	0.50	0.29–0.84
Guangzhou	191	64.88%	0.266	0.75	0.44–1.25
Kunming	180	76.18%	0.108	1.50	0.91–2.46
Yinchuan	188	69.79%	0.413	1.28	0.70–2.34
Ethnicity					
Others	97	62.51%	ref	ref	ref
Han nationality	1227	64.94%	0.276	1.37	0.77–2.42
Gender					
Male	495	62.54%	ref	ref	ref
Female	829	66.47%	0.787	1.05	0.72–1.55
Age (years)					
18–39	248	72.57%	ref	ref	ref
40–54	438	68.33%	0.184	0.70	0.41–1.19
55+	638	60.14%	0.008^a^	0.49	0.29–0.82
Household Income per month (yuan)					
Low/Medium	487	63.41%	ref	ref	ref
High	837	65.49%	0.292	1.21	0.84–1.75
Education					
Low/Medium	894	64.58%	ref	ref	ref
High	430	65.28%	0.209	0.80	0.57–1.13
Smoking spouse/partner					
No smoking spouse/partner	721	63.76%	ref	ref	ref
Have smoking spouse/partner	415	67.06%	0.799	1.07	0.65–1.75
No spouse/partner	188	65.22%	0.895	1.04	0.61–1.75
Noticed warning labels in last month					
Less than Often	1152	64.84%	ref	ref	ref
Often	172	64.35%	0.581	0.89	0.58–1.36
Number of smoking friends					
0	409	62.74%	ref	ref	ref
1	238	68.62%	0.565	1.18	0.66–2.11
2	264	67.69%	0.774	1.08	0.64–1.83
3	232	70.01%	0.515	1.19	0.70–2.01
4	69	46.87%	0.054	0.44	0.19–1.01
5	112	60.77%	0.425	0.79	0.43–1.43
Surveyed before/after new HWLs apply					
Before	1100	66.04%			
After	224	59.64%	0.044^a^	0.63	0.40–0.99

CI, Confidence interval; Significant levels are indicated as follows: *p < 0.05; **p < 0.001,***p < 0.0001

OR, Odd Ratio of adding more health information (0, refused/don’t know/ less health information/the same; 1, more health information)

Survey before/after new HWLs apply, Before (before 01/04/2012); After (after 01/04/2012)

^a^The percentage are weighted and the frequencies are unweighted

**Table 3:** Characteristics associated with whether non-smokers reported they support government including pictures as part of the health warning labels (*N* = 1324)

	*N*	Support pictures (%)	*p*	OR	95% CI
City					
Beijing	202	73.59%	ref	ref	ref
Shenyang	187	82.59%	0.170	1.77	0.78–4.05
Shanghai	196	70.22%	0.702	0.84	0.33–2.12
Changsha	180	90.86%	0.013^a^	3.22	1.28–8.07
Guangzhou	191	82.15%	0.159	1.69	0.81–3.50
Kunming	180	87.05%	0.039^a^	2.42	1.05–5.60
Yinchuan	188	76.07%	0.704	1.14	0.58–2.25
Ethnicity					
Others	97	75.46%	ref	ref	ref
Han nationality	1227	80.48%	0.155	1.62	0.83–3.17
Gender					
Male	495	78.65%	ref	ref	ref
Female	829	81.29%	0.555	0.90	0.64–1.27
Age (years)					
18–39	248	86.67%	ref	ref	ref
40–54	438	83.03%	0.899	0.97	0.56–1.65
55+	638	76.34%	0.145	0.71	0.44–1.13
Household Income per month (yuan)					
Low/Medium	487	80.19%	ref	ref	ref
High	837	80.14%	0.440	1.19	0.77–1.84
Education					
Low/Medium	894	79.40%	ref	ref	ref
High	430	81.99%	0.767	0.94	0.64–1.39
Smoking spouse/partner					
No smoking spouse/partner	721	77.46%	ref	ref	ref
Have smoking spouse/partner	415	87.97%	0.005^a^	2.03	1.24–3.33
No spouse/partner	188	78.07%	0.793	1.07	1.04–3.86
Noticed warning labels in last month					
Less than Often	1152	78.75%	ref	ref	ref
Often	172	90.33%	0.039^a^	2.00	1.04–3.86
Number of smoking friends					
0	409	78.85%	ref	ref	ref
1	238	78.84%	0.719	0.89	0.47–1.69
2	264	80.35%	0.643	0.88	0.49–1.55
3	232	81.11%	0.818	1.07	0.60–1.92
4	69	81.25%	0.748	1.14	0.50–2.62
5	112	84.17%	0.885	0.94	0.38–2.29
Surveyed before/after new HWLs apply					
Before	1100	79.88%	ref	ref	ref
After	224	81.31%	0.362	0.80	0.50–1.29

CI, Confidence interval; Significant levels are indicated as follows: *p < 0.05; **p < 0.001,***p < 0.0001

OR, Odd Ratio of supporting government including pictures as part of health warnings (0, refused/don’t know/neither support nor oppose/oppose/strongly oppose; 1, support/strongly support)

Survey before/after new HWLs apply, Before (before 01/04/2012); After (after 01/04/2012)

Support for including pictures, Respondents who reported they support government should include pictures as part of health warning labels

^a^The percentage are weighted and the frequencies are unweighted

## Discussion

In this study, we found that 12.2% of non-smokers noticed HWLs “often” in the last month. Respondents with a smoking spouse/partner and with 5 friends who smoke were more likely to notice the HWLs often. It is likely that non-smokers with smoking partners and friends had more opportunities to notice cigarette packs, and the HWLs in their daily life if their partner or friends smoked around them, and left cigarette packs out in the open. Although the proportion of respondents who noticed the HWLs was higher among those who completed the survey after the new HWLs were implemented (19.5% after vs. 10.4% before), the overall proportion who noticed the HWLs was still very low. It is possible that this increase in noticing was due to both the size of the text being increased, removing the English warning, and also an initial novelty effect of seeing a new HWL [17].

Sixty-four percent of respondents supported adding more health information to the HWLs, and 80.2% supported including pictures as part of HWLs. Another study in four cities (Beijing, Shanghai, Kunming and Yinchuan) of China in 2010 showed that among 357 adult non-smokers, 77.5% supported adding more health information to the HWLs, and 86.1% of non-smokers supported including pictures as part of HWLs [6]. The result from this study is also consistent with another study based on data from Jiangsu province in 2011, that showed that most people thought the text-only HWLs did not provide useful health information about the risks of smoking and that the HWLs should have more health information not only for smokers but also for non-smokers [18].

The main strength of this study is its representative sample of non-smokers from seven Chinese cities, most current studies are among smokers. The main limitations are the self-reported measures allowing the possibility for social desirability effects, and the cross-sectional design. However, a longitudinal design to evaluate pictorial HWL in China is not possible unless pictorial HWLs are implemented.

## Conclusion

The current HWLs in China are noticed by a minority of non-smokers and there is strong support to add more information and pictures to the HWLs. More effective HWLs could be particularly useful in China for educating non-smokers about the health risks of secondhand smoke. Additionally, educating non-smokers about the health risks of smoking with stronger HWLs may lead non-smokers to encourage family/close others to quit, and help prevent smoking uptake [19]. Together, with findings from previous studies, the current study suggests that the Chinese HWLs should be strengthened to at least meet the FCTC guidelines of 50% pictorial warnings. This study and others, from countries such as Canada, suggest that non-smokers strongly support including pictures as part of the HWLs, and that pictorial HWL more effectively communicate information about health risks [6, 8, 18–23].

## Abbreviations

CI: Confidence interval
HWLs: health warning labels
ITC: International tobacco control
OR: Odd ratio
WHO: World Health Organization
