# Erratum to: Noticing cigarette health warnings and support for new health warnings among non-smokers in China: findings from the International Tobacco Control project (ITC) China survey

**DOI:** 10.1186/s12889-017-4495-1

**Published:** 2017-06-19

**Authors:** Zejun Li, Tara Elton-Marshall, Geoffrey T. Fong, Anne Chiew Kin Quah, Guoze Feng, Yuan Jiang, Sara C. Hitchman

**Affiliations:** 1 Department of Basic and Clinical Neuroscience, Institute of Psychiatry, Psychology, and Neuroscience, King’s College London, London, England; 2 Social and Epidemiological Research Department, Centre for Addiction and Mental Health, London, Canada; 3 Department of Epidemiology and Biostatistics, Western University, London, Canada; 4 Dalla Lana School of Public Health, University of Toronto, Toronto, Canada; 5 Department of Psychology, University of Waterloo, Waterloo, Canada; 6 School of Public Health and Health Systems, University of Waterloo, Waterloo, Canada; 7 Ontario Institute for Cancer Research, Toronto, ON Canada; 8 Tobacco Control Office, Chinese Center for Disease Control and Prevention, Beijing, China; 9 Department of Addictions, Institute of Psychiatry, Psychology, and Neuroscience, King’s College London, London, England; 10 UK Centre for Tobacco and Alcohol Studies, Nottingham, UK; 11 Addictions Department, Addictions Sciences Building, Institute of Psychiatry, Psychology and Neuroscience (IoPPN), King’s College London, 4 Windsor Walk, Denmark Hill, London, SE5 8BB England

## Erratum

Following publication of this article [1], it has come to our attention that the correct full name of the second author is “Tara Elton-Marshall”. The original version of the article has been revised to reflect this.
